# Internet Addiction among Young Adult University Students during the COVID-19 Pandemic: The Role of Peritraumatic Distress, Attachment, and Alexithymia

**DOI:** 10.3390/ijerph192315582

**Published:** 2022-11-24

**Authors:** Eleonora Marzilli, Luca Cerniglia, Silvia Cimino, Renata Tambelli

**Affiliations:** 1 Department of Dynamic, Clinical & Health Psychology, Sapienza University of Rome, 00185 Rome, Italy; 2 Faculty of Psychology, International Telematic University Uninettuno, 00185 Rome, Italy

**Keywords:** university students, COVID-19, internet addiction, attachment, alexithymia

## Abstract

The literature focused on the effect of the COVID-19 pandemic on young adult university students’ mental health shows a significant increase in psychopathological symptoms and Internet Addiction (IA). The key role played by attachment and alexithymia has also been suggested, but no study has explored the possible dynamic relationship between these variables. We recruited a sample of *n* = 410 young adult university students online. We assessed the attachment to parents and peers (through IPPA), alexithymia (through TAS-20), peritraumatic distress symptoms due to COVID-19 (through CPDI), and IA (through IAT). The results showed that the relationship between the attachment to mothers and IA was partially mediated by alexithymia and by the serial mediation of alexithymia and peritraumatic distress, whereas the influence of the attachment to fathers on IA was fully mediated by peritraumatic distress. The direct effects of the attachment to peers on alexithymia, peritraumatic distress, and IA were all significant, as were the indirect paths via the simple mediation of both alexithymia and peritraumatic distress and the multiple serial mediation of alexithymia and peritraumatic distress. Our findings suggested that the relationship between attachment, alexithymia, and psychopathological risk is dynamic in predicting IA during the pandemic among young adult university students and that the different attachment figures exert a peculiar contribution to these processes.

## 1. Introduction

Since the beginning of the COVID-19 pandemic in March 2020, the restrictive measures taken worldwide to reduce its dramatic spread (e.g., physical distancing, limited mobility, lockdowns, and remote learning/working) have had a significant impact on the daily routine of people, resulting in a worrying increase in psychopathological symptoms among the general population [1]. Moreover, due to containment measures, a considerable increase in internet use has been shown [2], primarily to cope with social isolation and to communicate with others and to search for information more easily [3,4]. Despite these potentially useful effects of internet use during the COVID-19 pandemic, the scientific literature had also shown that some people tended to use the internet in a problematic and excessive way as a strategy to cope with the emotional difficulties and psychological suffering resulting from the pandemic [5], to the point of developing Internet Addiction (IA) symptoms [6,7]. To date, there are no official diagnostic criteria for IA, and its definition is still debated [8]. The revision of the fifth edition of the Diagnostic and Statistical Manual of Mental Disorders (DSM-5) [9] has only inserted an internet-related condition in Section 3 (i.e., the Internet Gaming Disorder), defining it in terms of a persistent pattern of behavioral addiction associated with loss of control in internet gaming and significant functional disruption [10]. The international literature has proposed several terms to refer to the maladaptive use of the internet (e.g., problematic internet use and pathological internet use) [11], all evidencing specific common features, including the uncontrollable and obsessive–compulsive use of the internet, the pressing need to spend even more time online, the presence of irritability when the internet cannot be used, craving, and interpersonal impairment [12]. In this study, we have chosen to use the term IA in line with the conceptualization of Young [13], who, highlighting its characteristics of compulsiveness, defined this phenomenon as the excessive and uncontrollable use of the internet, which leads to important consequences with regard to emotional, behavioral, and social functioning [14].

In this scenario, university students have been one of the most vulnerable populations with respect to the negative effect of the pandemic on both the psychopathological risk [15,16] and IA [17,18]. Indeed, the government of Italy and most states around the world recommended the remote switching of academic activities, including distance-learning classes, access to online books, the shift to online assessments, and the cancellation of in-person graduation ceremonies. Many students have shown difficulties in adapting functionally to these challenges, manifesting more concerns about their academic performances [19] and an increase in post-traumatic stress disorder-like symptoms, defined in terms of peritraumatic distress symptoms (e.g., anxiety and depressive symptoms, specific phobia, avoidance behaviors, and compulsive behaviors) [20,21]. Notably, the pre-pandemic studies widely showed the comorbidity between IA and other psychological problems, especially depression and anxiety symptoms [22,23]. Recently, the same associations have also been found immediately after the outbreak of COVID-19 [5,24] and in the later stages of the pandemic [25]. Although it has been underlined that this relationship may be reciprocal and bidirectional [26], recent researchers have suggested considering IA as a resulting strategy to cope with the suffering underlying these other psychopathological difficulties [27]. In this context, it is important to note that the alarming impact of COVID-19 and its related restrictions on people’s psychological well-being was accompanied by a significant difficulty and reduction in the accessing of mental health services during the first wave of the pandemic [28], predisposing people to greater vulnerability in coping with the negative effects of the pandemic. Longitudinal studies have also evidenced that a few months after the pandemic’s start, with the end of the so-called “first wave”, people showed adequate abilities to adapt to COVID-19-related environmental conditions and showed reduced psychopathological symptoms [29,30]. However, with the advent of the so-called “second wave”, there was a significant further rise in peritraumatic distress symptoms [31,32], supporting the importance of implementing studies on the psychological impact of COVID-19 as the pandemic continues.

A longitudinal study by Zhao et al. [33] has underlined that the short- and long-term effects of the pandemic on university students’ psychological well-being have also included a significant increase in IA prevalence rates. This may be due to the fact that, as evidenced above, the changes in academic life resulting from the COVID-19 restrictions have led to a substantial increase in the daily time spent on the internet. Since the period before the pandemic, the previous literature has widely highlighted that a high amount of time spent online is a risk factor for the onset of IA [34]. Consequently, the great exposure to internet use that university life entails, promoting free and unrestricted internet access and actively encouraging students to use the internet for the execution of academic tasks, may lead university students to an increased risk of IA [35]. However, several other reasons, beyond those strictly related to academic activities, can make university students a particularly at-risk group for the developing of IA, including the large amount of free and unstructured time available to them; possible difficulties in adapting to their new academic life and in establishing new friendships, which may expose them to more active online relationship-seeking behavior; the use of the internet as an escape route from the sources of stress related to passing exams [36]. Moreover, during young adulthood, the management of time spent on the internet is no longer under parental control and becomes an individual responsibility of the university students [37,38]. In the specific context of the pandemic, social distancing measures have also led to limited opportunities to socialize and establish face-to-face relationships, which in turn may increase the risk of developing IA [39]. Furthermore, the research has underlined that being a young adult university student represents an adjunctive risk factor for the negative effect of the pandemic on these processes. Indeed, a higher psychopathological risk due to COVID-19 among young adults has been reported [40], with a higher prevalence among females [41]. At the same time, epidemiological studies have shown that this population group is among the most frequent users of the internet, both before [42] and from the beginning of the pandemic [18]. Beyond this, some phase-specific developmental characteristics also contribute to their greater vulnerability. Specifically, in accordance with Erikson’s psychosocial theory [43], young adulthood (youths aged approximately between 18 and 25 years) live the so-called “Intimacy vs. Isolation” psychosocial crisis in which the major conflicts are related to establishing loving relationships and not feeling isolated from other people. Other authors have underlined that young adults must undertake important developmental tasks (e.g., the redefinition of relationships with parents, peers, and society; the acquisition of identity and autonomy), which the use of the internet can help them to face [44]. However, many studies have shown that these phase-specific characteristics put them at a higher risk of the onset of IA [45,46] and that the pandemic has further exacerbated this increased risk [47,48], especially among female youths [49]. In this context, the Self-Medication Hypothesis [50] considered addictive behaviors as a maladaptive strategy aimed at obtaining relief from painful emotions or from experiencing/controlling emotions. In accordance with this theoretical model, recent studies [5,51] have evidenced that the psychopathological symptoms due to COVID-19 significantly predicted higher levels of IA. These studies suggested that the increase in the peritraumatic distress levels due to the COVID-19 pandemic is associated with higher IA symptoms among university students as a strategy to cope with the negative emotions resulting from the COVID-19 pandemic and its related restrictions. Given the clinical and social relevance of the phenomenon, the implementation of the research on the possible factors that may mitigate or exacerbate the effect of young adult university students’ mental health on their increased risk of IA is necessary. In this context, clinicians and researchers rooted in the developmental psychopathology theoretical framework [52,53] have widely supported the importance of considering the role played by the complex interplay between relational variables and individual vulnerabilities in studying the underpinning mechanisms of the psychopathological difficulties among young adults. 

### 1.1. The Role of Attachment to Parents and Peers

Among relational influences, the quality of attachment to parents and peer groups has been suggested to have a central risk and/or protective contribution to the effects of stressful life events on young adult university students’ psychological well-being [54,55], as well as on the subsequent risk of IA [56]. Specifically, the attachment system is activated in response to adverse life events [57], such as the COVID-19 pandemic, leading the individual to use related emotional reactions and stress regulation strategies. In this context, Shumaker and Brownell [58] have postulated that social support is a crucial variable in sustaining an individual’s psychological well-being, especially in coping with stressful life events. The authors defined social support in the terms of ‘an exchange of resources between at least two individuals perceived by the provider or recipient to be intended to enhance the well being of the recipient’ and which may derive from different significant sources, especially parents and friends [58]. Attachment theory [59] pointed out that secure attachment is a prerequisite for an individual’s ability to involve himself/herself in a social network necessary for the creation of socially supportive relationships. Previous studies have shown that attachment security is significantly associated with the activation of adaptive coping strategies in response to traumatic events, such as seeking comfort and emotional support from parents and friends [60,61]. Consequently, given that the COVID-19 pandemic can be qualified as a traumatic event potentially triggering post-traumatic stress disorder-like symptoms [62], the quality of individual attachment can direct the coping strategies to face this experience [63]. On the other hand, extensive research has shown significant associations between insecure attachment and psychopathological symptoms throughout the lifespan, including young adulthood [64,65], which in turn are associated with a higher risk of developing IA [66,67]. Recently, studies by Moccia et al. [68] and Muzi et al. [69] have reported the same association between the insecure attachment and the psychopathological symptoms resulting from the COVID-19 pandemic. At the same time, several studies have shown that a poor quality of relationships with parents and peers has a crucial influence on addictive behaviors among young adult populations [14] and university students [46], including IA [45,70]. A recent study by Trumello et al. [71] showed that there were also significant associations between attachment insecurity and IA during the COVID-19 pandemic. Overall, this body of research suggests that young adult university students may excessively use the internet as a strategy to cope with the distress resulting from insecure relationships with their attachment figures [72,73] and to seek social support from the virtual world [74]. Interestingly, the possible mediation role played by the young adults’ psychopathological symptoms on the relationship between the quality of their relationships with parents and peers and the IA levels has recently been suggested [46,48,75]. However, despite this evidence, to date no study has focused on university students in exploring these processes during the COVID-19 pandemic.

### 1.2. The Dynamic Relationship with Alexithymia

From the beginning of the pandemic, the research focused on the individual variables that may link to a higher vulnerability in response to the pandemic and its related restrictions has evidenced the key role played by alexithymic traits [76,77]. Alexithymia is defined as a difficulty in identifying and recognizing one’s own feelings, with externally oriented thinking and a deficit in regulating emotions [78]. Consequently, university students who have difficulties in identifying, expressing, and regulating their feelings may experience higher emotional pain when facing stressful events, such as COVID-19. As a result, they may the use the internet in a maladaptive way to regulate their emotions and obtain social support [79]. In line with this, significant associations between alexithymia and both the psychopathological symptoms resulting from COVID-19 [51,76,77] and IA [80] have recently been shown. Regarding possible sex differences, the recent study by Lyvers et al. [81] reported higher alexithymia among male young adults, although the study by Wang et al. [82] found no significant differences between males and females. Notably, the findings of previous studies in this field suggested the possibility of a complex relationship between alexithymia, attachment, and psychological well-being. Specifically, insecure attachment represents one of the main risk factors for the onset and maintenance of emotional regulation problems [75], predisposing individuals to higher alexithymic traits [83,84], and exerting an additional risk influence over the effects of alexithymia on psychopathological symptoms [85] and IA [86]. Moreover, recent studies have suggested that alexithymia is a significant mediator in the relationship between the quality of attachment and both the psychopathological symptoms [84] and IA [73,84]. 

### 1.3. The Present Study

A growing body of research focused on university students’ psychological well-being during the COVID-19 pandemic has evidenced a worrying increase in peritraumatic distress symptoms [20,21] and IA levels [17,18], especially among young adults. In this context, a key role played by the quality of attachment and alexithymia on both the psychopathological risk resulting from COVID-19 [51,69,76,77] and IA has been reported [71,80,87]. Interestingly, in line with the developmental psychopathology perspective, previous studies have underlined the complex interplay between these variables. Specifically, alexithymia has been shown to be a significant mediator in the relationship between young adults’ attachment to parents and peers and psychopathological symptoms [85], as well as in the relation to the psychopathological symptoms specifically related to the COVID-19 pandemic [87]. However, no study has yet specifically focused on university students. On the other hand, it has recently been suggested that young adults’ alexithymic levels mediate the relationship between the quality of attachment and the peritraumatic distress due to COVID-19 [88]. In line with this previous evidence, a possible serial mediation of young adult university students’ alexithymia and peritraumatic distress symptoms resulting from COVID-19 on the relationship between attachment to parents and peers and IA could be suggested, but to date, this complex relationship has remained unexplored. 

We recognize that studies using longitudinal research designs represent the elective methodology for assessing causal relationships between variables [89]. Nevertheless, some authors have suggested that if there is previous literature that has demonstrated the causal role of relationships between the variables under study, this can be used to define the order of the variables [90,91]. Testing their possible causal relationships also used cross-sectional designs [92].

Based on these premises and literature gaps, this study aimed to verify: (a) the possible sex differences in the levels of peritraumatic distress symptoms, alexithymia, and IA. Based on the previous literature [41], we hypothesized that higher peritraumatic distress symptoms could be found among females. Regarding possible sex differences in alexithymia and IA, the past studies reported conflicting results [81,82]. Consequently, we aimed to add new knowledge regarding the possible role played by the sex of the young adult university students in these processes. (b) The study aimed to verify whether young adult university students’ alexithymia and their peritraumatic distress symptoms due to COVID-19 simply and serially mediated the relationship, respectively, between the attachment to mothers, fathers, and peers, and IA levels, while controlling for relevant covariates. Previous evidence has shown that both attachment [68,69], alexithymia [71,80], and peritraumatic distress symptoms due to COVID-19 [5,51] significantly predicted IA but also that attachment significantly predicted alexithymia [83,84,88], which in turn predicted psychopathological distress due to COVID-19 [51,76,77]. Based on this, this study hypothesized that alexithymia and peritraumatic distress symptoms will be identified as mediating the relationship between young adult university students’ attachment and IA levels. Furthermore, we hypothesized that the mediation role played by these variables varied on the basis of the different attachment figures (i.e., mothers, fathers, and peers).

## 2. Materials and Methods

### 2.1. Procedure

The study was conducted between 15 November 2020 and 15 March 2021, during the Italian second wave of COVID-19. Before its start, the study was approved by the Ethical Committee of the Department of Dynamic and Clinical Psychology at Sapienza University of Rome (protocol N. 809/2020), in accordance with the Declaration of Helsinki. We recruited *n* = 578 university students. The participating universities were selected based on the researchers’ affiliations (i.e., the University of Rome “Sapienza” and the “International Telematic University Uninettuno”, in the center of Italy). All the participants lived in Rome. The sample was recruited online. Specifically, a recruitment message was posted on the researcher’s Facebook page and via notices posted on online psychology research websites. No compensation was provided for the participants. After obtaining the university students’ written informed consent, each subject completed self-report instruments (described below) through an anonymous online survey. First, the participants completed an ad hoc questionnaire assessing inclusion/exclusion criteria (i.e., age, the presence/absence of mental and/or physical diagnoses and/or ongoing psychological and/or psychiatric treatment), and relevant sociodemographic variables (i.e., sex, family structure, romantic relationship status, living setup, and household income). Moreover, the participants were asked to recall and report their estimated hours per day spent online for leisure activities/entertainment and study/academic activities before the pandemic and the hours per day spent online since the beginning of the COVID-19 pandemic. Then, self-report instruments for the assessment of attachment to parents and peers, alexithymia, peritraumatic distress symptoms due to COVID-19, and IA were administered.

### 2.2. Measures

For the assessment of the variables under study, all the emerging adult university students who agreed to take part in the study filled out the following self-report questionnaires: the Inventory of Parent and Peer Attachment (IPPA) [93,94], the COVID-19 Peritraumatic Distress Index (CPDI) [95,96], the Toronto Alexithymia Scale (TAS-20) [97,98], and the Internet Addiction Test (IAT) [10,99]. The IPPA [93] is a self-report questionnaire assessing the individual’s perception of the quality of their relationships with parents (with mothers and fathers separately) and peers. It is composed of three parts; the first is for the assessment of the attachment to mothers (which included 28 items; e.g., “Talking over my problems with my mother makes me feel ashamed or foolish”); the second is for the assessment of the attachment to fathers (which included 28 items; e.g., “My father can tell when I’m upset about something”); and the last is for the assessment of attachment to peers (which included 25 items; e.g., “When we discuss things, my friends care about my point of view”). The items are rated on a 5-point Likert scale, and higher scores indicate greater attachment security. The Italian validation [94] showed good psychometric proprieties (the Cronbach’s alpha ranged from 0.62 to 0.90). In the present study, the Cronbach’s alpha for the total score of attachment to mothers was 0.78; for attachment to fathers it was 0.77; and for attachment to peers it was 0.86. The CPDI [95,96] is a self-report instrument composed of 24 items assessing the psychological distress resulting from COVID-19 related to anxiety/depressive symptoms, avoidance and compulsive behaviors, phobic thinking, and impairment in social functioning (e.g., “Compared to usual, I feel more nervous and anxious”, “I collect information about COVID-19 all day. Even if it’s not necessary, I can’t stop myself”). Each item is measured on a 5-point Likert scale response format, ranging from 0 (‘not at all’) to 4 (‘extremely’). The total score ranges from 0 to 100, with higher scores indicating more psychological distress. Specifically, in line with the CPDI’s cutoff [95] and with the Italian validation [96], scores below 28 indicate no distress, between 28 and 51 indicate mild to moderate distress, and above 51 severe distress. The CPDI has shown good internal coherence [95,96], which in this study was also adequate (Cronbach’s alpha = 0.87). The TAS-20 [97,98] is a self-report questionnaire composed of 20 items assessing the alexithymic level. The items are rated on a 5-point Likert scale, ranging from 1 (‘strongly disagree’) to 5 (‘strongly agree’). Specifically, the scale is composed of three factors reflecting, respectively, the ability to recognize emotions (e.g., “It is difficult for me to find the right words for my feelings”; the ability to describe verbally one’s own emotions (e.g., “I am often confused about what emotion I am feeling”); and the tendency of externally oriented thinking (e.g., “I prefer to analyze problems rather than just describe them”). The total score used in this study results from the sum of the scores of the three factors. Based on the recommended TAS-20 cutoff scores [97,98], scores below 50 are indicative of no alexithymia, between 51 and 60 indicate borderline alexithymia level, and above 61 indicate alexithymia. The TAS-20 showed good internal consistency and test–retest reliability (the Cronbach’s alpha of the total score is 0.87 in this study). The IAT [10,99] is a self-report questionnaire assessing the severity of Internet Addiction. It is composed of 20 items measured on a 5-point Likert scale assessing the characteristics and behaviors associated with the compulsive use of the internet (including compulsivity, escapism, and dependency). Specifically, the IAT is composed of six factors: Salience (e.g., “How often do you block out disturbing thoughts about your life with soothing thoughts of the Internet?”); Excessive Use (e.g., “How often do you find that you stay online longer than you intended?”); Neglect of Work (e.g., “How often do your grades or school work suffer because of the amount of time you spend online?”); Anticipation (e.g., “How often do you check your email before something else that you need to do?”); Lack of Control (e.g., “How often do others in your life complain to you about the amount of time you spend online?”); and Neglect of Social Life (e.g., “How often do you prefer the excitement of the Internet to intimacy with your partner?”). However, through this instrument, it is possible to obtain a total score, used in this study, which allows an assessment of the presence and the severity of the IA. Higher scores are indicative of a higher severity of the compulsive use of the internet, with a maximum score of 100. Specifically, in line with the Italian validation [99], scores from 0 to 30 indicate average users with complete control of their internet use; scores from 31 to 49 are indicative of excessive internet use and moderately addicted users; and scores from 80 to 100 indicate severely addicted users. The scale showed very good internal consistency, with the Cronbach’s alpha = 0.91 in this study.

### 2.3. Statistical Analyses

First, preliminary statistical analyses were conducted (i.e., reliability of the measures, frequencies, percentages, and mean scores). Then, given the ordinal level of the measurement of the time spent online by university students before and from the beginning of the COVID-19 pandemic, and the not-normal distribution of the variables (Shapiro–Wilk test = *p* < 0.001), the nonparametric Wilcoxon signed-rank test was used to assess the possible change between the recall of the estimated average hours per day spent online before the pandemic and the average hours per day spent online from the beginning of the COVID-19 pandemic by young adult university students for leisure activities/entertainment and academic activities. To verify the possible differences between males and females in peritraumatic distress symptoms, alexithymia, and IA, multivariate analyses of variance (MANOVAs) were conducted, with young adult university students’ sex as the independent variable and the scores of IPPA, TAS, CPDI, and IA as the dependent variables. Then, to determine the initial correlations between the study variables, Pearson’s correlation analyses were carried out. Based on the significant correlations and the theoretical model, mediation analyses were performed to verify whether young adult university students’ alexithymia and peritraumatic distress levels simply and serially mediated the effect of, respectively, attachment to mothers, fathers, and peers on IA, while controlling for significant covariates. We used Hayes’s [100] PROCESS macro (Model 6) to conduct mediation analyses, evaluating indirect effects with 95% bias-corrected confidence intervals (CIs), based on 10,000 bootstrap samples. All analyses were performed using SPSS software, Version 27 (IBM, Armonk, NY, USA).

## 3. Results

### 3.1. Sample Characteristics

For the aim of this study, we excluded university students who were not aged between 19 and 25 years (*n* = 42); who had mental and/or physical diagnoses (*n* = 35); who were following a psychological and/or psychiatric treatment (*n* = 27); and who had not completed the assessment procedure (*n* = 49). The final sample consisted of N = 410 young adult university students (71.2% females; Mage = 23.41, SD = 1.72), living in Italy. Most of them (74.1%) were in intact family groups. Sixty-two point two percent of the young adult university students were single, 81.5% lived with family members, and most often (37.6%) reported a household income between EUR 55,001 and 75,000 per year. Table 1 reports the complete description of the sample demographic characteristics.

Based on the TAS-20 cutoff [97,98], 44.4% of the sample showed no alexithymia, 28.3% reported borderline alexithymia levels, and 27.3% had alexithymia. Moreover, according to the Italian validation of the IAT [99], 75.9% of young adult university students reported complete control of their internet use, 22.9% showed excessive internet use, and N = 4 (1.2%) had IA. Finally, in accordance with the CPDI cutoff [95,96], we found that 23.7% of the sample showed no peritraumatic distress symptoms due to COVID-19, 54.9% reported mild/moderate distress, and 21.5% of young adult university students showed severe peritraumatic distress due to the pandemic.

### 3.2. Differences in University Students’ Time Spent Online between before and from the Beginning of the COVID-19 Pandemic

Table 2 shows the descriptive statistics of the recall of the estimated time spent on the internet by university students for leisure activities/entertainment and for study/academic activities before and since the beginning of the COVID-19 pandemic.

The results of the Wilcoxon signed-rank test showed that the median of the time spent online by young adult university students on leisure activities/entertainment during the COVID-19 pandemic was significantly higher than those spent before the COVID-19 pandemic (Z = −13.93, *p* < 0.001). At the same time, the median of the time spent online for study/academic activities during the COVID-19 pandemic was statistically significantly higher than the median of time spent online for academic activities before the COVID-19 pandemic (Z = −13.92, *p* < 0.001). 

### 3.3. Sex Differences in Peritraumatic Distress Due to COVID-19, Alexithymia, and Internet Addiction

The results of the MANOVA showed an overall significant difference between males and females (F_3406_ = 11,003, *p* < 0.001, Hotelling T^2^ = 33,048). The results of the univariate effects, as reported in Table 3, showed that the female university students showed significantly higher peritraumatic distress symptoms due to COVID-19 than the males (*p* < 0.001). Conversely, there were no significant differences in the levels of alexithymia and IA between the male and female young adults (all *p* > 0.05).

### 3.4. University Students’ Alexithymia and Peritraumatic Distress Symptoms Due to COVID-19 as Mediators of the Relationship between Attachment to Parents and Peers and Internet Addiction

First, Pearson’s correlation analyses to investigate the significant correlations between the measured variables were conducted. The results showed that the IAT scores were significantly and positively associated with the CPDI and TAS scores, and negatively associated with the scores of the IPPA (attachment to mothers, fathers, and peers). Moreover, both the CPDI and the TAS scores were more significantly and negatively associated with the IPPA scores than with each other (Table 4).

**Figure 1:**
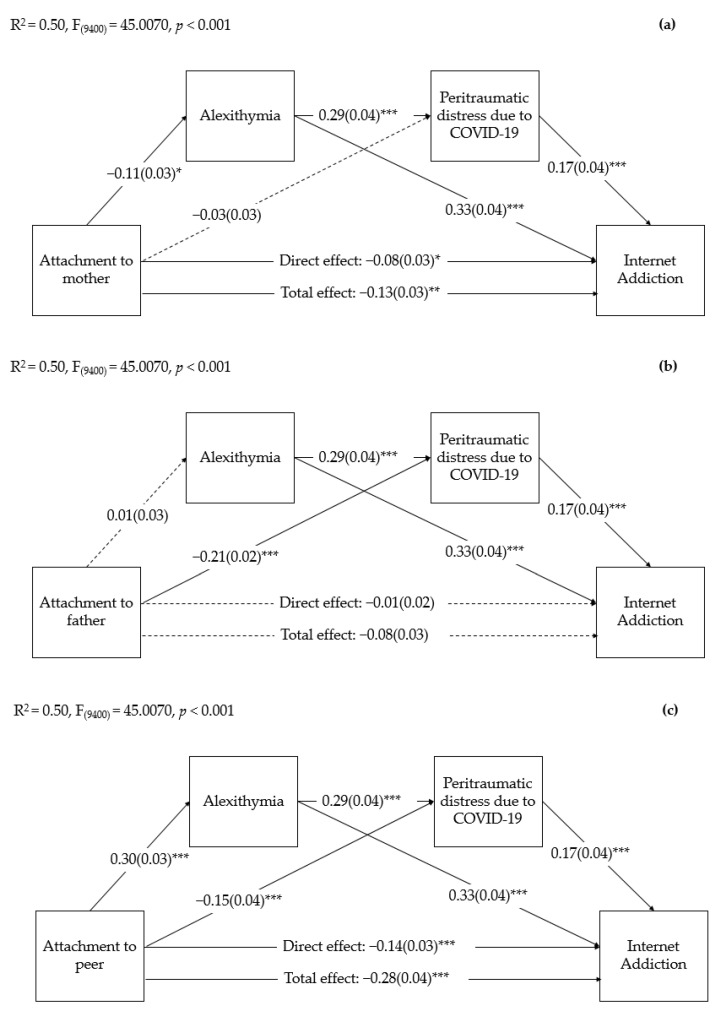
Serial mediation of young adult university students’ alexithymia and peritraumatic distress due to COVID-19 on the relationship between attachment to mothers and Internet Addiction (**a**), attachment to fathers and Internet Addiction (**b**), and attachment to peers and Internet Addiction (**c**). Coefficients shown are standardized path coefficients. Dotted lines represent non-significant parameters. * *p* < 0.05, ** *p* < 0.01, *** *p* < 0.001.

Based on the significant correlations, we carried out mediation analyses to explore whether the young adult university students’ levels of alexithymia and their levels of peritraumatic distress due to COVID-19 separately and serially mediated the relationship between the attachment to their mothers, fathers, and peers and the levels of IA. The university students’ age and sex significantly correlated to many of the study variables and were included as covariates in the mediation analyses. Moreover, given the significant increase that we found in time spent online since the beginning of the pandemic for both leisure activities/entertainment and study/academic activities, we also included these variables as covariates. Finally, for each mediation model we considered, respectively, the young adults’ attachment to their mothers, fathers, and peers as additional covariates. As can be seen in Figure 1a, the results of the mediation analyses showed that the total and direct effects of the young adult university students’ attachment to their mothers on their levels of IA were significant. The direct effect on alexithymia of the attachment to their mothers was also significant, but on the peritraumatic distress levels due to COVID-19, it was not. In addition, alexithymia, as the first mediating variable, significantly predicted the young adults’ peritraumatic distress levels, as the second mediating variable. The direct effects of alexithymia and peritraumatic distress on the IA levels were also significant.

Regarding the indirect effects, Table 5 shows that the indirect paths via the single mediation of alexithymia and via the multiple serial mediation of alexithymia and peritraumatic distress due to COVID-19 were statistically significant. Conversely, the single mediation of peritraumatic distress levels was not significant. Regarding attachment to fathers, as can be seen in Figure 1b, the total and direct effects of attachment to fathers on the levels of young adult university students’ IA were not significant. Moreover, the direct effect of attachment to fathers on alexithymia was also not significant. Attachment to fathers significantly directly predicted the peritraumatic distress levels due to COVID-19. Regarding the indirect effects, as can be seen in Table 5, the indirect path via the single mediation of peritraumatic distress was significant, whereas the single mediation of alexithymia and the multiple serial mediation of alexithymia and peritraumatic distress were not. Finally, the direct and total effects of attachment to peers on levels of IA and the direct effect of attachment to peers on alexithymia were significant (Figure 1c). Table 5 shows that the indirect paths via the simple mediation of both alexithymia and peritraumatic distress, as well as the multiple serial mediation of alexithymia and peritraumatic distress, were significant.

## 4. Discussion

The recent literature that has been interested in investigating the impact of the COVID-19 pandemic on young adult university students’ psychological well-being has reported a worrying increase in psychopathological symptoms [20,21] and IA [17,18]. Consequently, this study aimed to further increase the knowledge on the possible relevant risk and/or protective factors that may exacerbate or mitigate the effect of the pandemic on the psychopathological risk and the IA risk among young adult university students. Specifically, we based our work on the developmental psychopathological framework [52,53] that considers young adults’ psychopathologic outcomes as the result of a complex interaction between risk and/or protective factors of different natures, evidencing especially the key role played by relational factors and emotional regulation difficulties. In the specific context of the pandemic, previous studies have underlined that alexithymia significantly predicted the peritraumatic distress level [88,101], which in turn predisposed individuals to a higher risk of IA [87,102]. The predictive role played by the quality of attachment on peritraumatic distress, alexithymia, and IA has also been shown [68,69,71,80,88]. Interestingly, the past studies underlined that young adults’ peritraumatic distress due to COVID-19 significantly mediated the association between alexithymia and IA [87], and alexithymia has been shown to be a significant mediator in the relationship between young adults’ attachment to parents and peers and the peritraumatic distress levels resulting from the pandemic [88]. However, although these studies were focused on young adult populations, no study has yet explored these processes among young adult university students. Moreover, although previous evidence may suggest the presence of a serial mediation of alexithymia and peritraumatic distress in the relationship between attachment and IA, this possible complex relationship has yet been unexplored.

### 4.1. Preliminary Findings

The results of the descriptive statistics on psychological variables showed that approximately 55% of the sample had a moderate level of peritraumatic distress due to the COVID-19 pandemic, and 21.5% of them reported severe distress. These rates of prevalence are in line with the study by Jiménez et al. [103] on a sample of the general population that has also shown that young adults experienced more distress than those of an older age. The study by Mohamed et al. [21] also found clinical levels of peritraumatic distress symptoms among university students during the first wave of the COVID-19 pandemic, but with lower rates. This could be due to the fact that our study was conducted more than a year from the beginning of the pandemic, during the so called “second wave”, confirming the literature that has underlined a deterioration in people’s mental health as the pandemic continues [31]. Regarding IA, we found that 22.9% of university students were moderately addicted, whereas 1.2% of them were severely addicted users. Epidemiological studies before the pandemic outbreak reported lower prevalence rates of IA among university students (approximately 11%) [104,105]. Our findings are in line with the studies conducted during COVID-19 that have reported prevalence rates of IA between 21% and 27% among young adult students [106,107]. This increase in the rates of IA prevalence during the COVID-19 pandemic could be due to the parallel increase in internet usage [6,20], as our study has also evidenced. Indeed, we found a significant increase in the time spent online by young adult university students from the beginning of the pandemic for both leisure activities/entertainment and study/academic activities. This is in line with the findings of several studies suggesting the significant contribution of time spent online to the onset of IA [34]. Finally, 28.3% of our sample showed subthreshold alexithymia levels, whereas 27.3% had alexithymia, which is in line with past studies conducted on young adult university students [108]. Regarding the possible sex differences, we only found that females reported higher peritraumatic distress levels resulting from COVID-19 than males, which is in line with our hypotheses and with previous studies in the field [41]. Conversely, we found no significant sex differences in IA and alexithymia levels. However, the research produced conflicting results on possible sex-related differences in these variables among young adults. Indeed, although some studies have reported higher rates of prevalence among males [81], our study is in line with the recent body of research that evidenced no sex differences [82]. On these contrasting findings of sex differences in IA, previous studies have suggested that these could be related to the presence of sex-related differences in the underpinning etiopathogenetic factors [109]. In particular, some studies have shown that higher levels of impulsivity and anxiety/depressive symptoms may expose, respectively, males and females to a higher risk of IA [110,111]. Different developmental trajectories of IA between males and females were also reported, with males being at a higher risk for IA during adolescence, while females reported a more rapid increase later in time [112]. Regarding alexithymia, brain structure and chemistry, as well as hormonal balance, are factors that have been shown to play a key role in sex-based differences in alexithymic levels [113]. Moreover, several authors have suggested that females tend to manifest better ability in processing and interpreting their own and others’ emotions [114] and that males may have reported higher alexithymic traits than females due to sex-related social pressure [115]. On the other hand, other studies have suggested that this female advantage may also result in a higher risk of developing affective disorders [116,117] and that the relationship between alexithymia and difficulties in coping with life event stressors is more relevant and stronger among females, especially during adolescence and young adulthood [118,119].

### 4.2. The Simple and Serial Mediation of Alexithymia and Peritraumatic Distress on the Relationship between Attachment (to Parents and Peers) and IA

Our last aim was to explore the complex relationship between the study variables. Overall, our results confirmed our starting hypotheses. Specifically, our preliminary correlational analyses found significant associations between IA and young adult university students’ peritraumatic distress symptoms, attachment to parents and peers, and alexithymia. Moreover, the attachment quality was significantly associated with alexithymia and peritraumatic distress, which in turn were associated with each other, supporting the possibility of a complex interplay between them. The results of the subsequent mediation analyses went further in this direction. Specifically, regarding direct effects, we found that young adult university students’ peritraumatic distress symptoms were significantly associated with IA levels. In this context, previous studies have defined IA as a maladaptive strategy used by the individual to cope with the psychopathological symptoms resulting from stressful life events [120]. Coherently, in the specific context of the COVID-19 outbreak, internet addictive behaviors have been conceptualized as a dysfunctional way to regulate the increased personal distress resulting from the pandemic [51,76], as our study also suggested. Moreover, alexithymia, as the first mediator, was significantly associated with both peritraumatic distress levels (as the second mediator) and IA. In this regard, extensive literature has evidenced that individuals with high levels of alexithymia manifested difficulties in the processing and regulation of their emotions [121,122], which exposed them to a higher risk of developing psychopathological symptoms when facing stressful experiences [88,123]. In the specific context of the pandemic, recent studies have also reported that alexithymia significantly predicted the psychopathological symptoms due to COVID-19 [51,76,77], but this is the first study that specifically evidenced this association among young adult university students. Moreover, as suggested by Zdankiewicz-Scigala and Scigala [124], due to difficulties in emotional regulation individuals with high alexithymic traits tend to cope with negative emotional states behaviorally rather than cognitively. In line with this, it has been suggested that IA is a strategy for maintaining emotional stability [125,126], especially when coping with stressful life events [21,127], including the COVID-19 pandemic [5,51,87]. Regarding the role played by the quality of attachment to parents in predicting IA, our results confirmed their crucial contribution and the hypothesis considering IA as a maladaptive strategy to cope with the suffering arising from perceived unsatisfactory parent–child relationships and to satisfy, in the virtual world, the need for emotionally caring relationships [71,128]. More specifically, our findings evidenced a complex reality, showing a specific contribution played by the quality of the relationship with the different attachment figures. Indeed, young adulthood represents a phase of life in which the individual tends to gain more independence from parental figures and in which extrafamilial relationships assume increasingly crucial importance in the young adults’ emotional life [129]. However, the relationship with mothers and fathers continues to assume a fundamental role and contributes to the psychological, emotional, and affective well-being of the individual [45,130,131]. In line with this, we found that the young adult university students’ attachment to their mothers was significantly associated with their levels of alexithymia and IA, but the association with peritraumatic distress levels due to COVID-19 was not significant. Notably, the indirect paths via the single mediation of alexithymia and via the multiple serial mediation of alexithymia and peritraumatic distress due to COVID-19 were statistically significant, whereas the single mediation of peritraumatic distress levels was not. Conversely, attachment to fathers was significantly and directly associated with peritraumatic distress levels due to COVID-19, but its direct effects on young adult university students’ alexithymia and IA levels were not significant. Moreover, the indirect path via the single mediation of peritraumatic distress was significant, whereas the single mediation of alexithymia and the multiple serial mediation of alexithymia and peritraumatic distress were not. These findings supported the past evidence on the key role exerted by insecure attachment to parents on the development of IA during young adulthood [132], as well as among university students. Coherently, a young adult university student who perceived their relationship with their parents as characterized by a lack of closeness, warmth, and emotional availability may tend to excessively use the internet to seek emotional and social support in online relationships [72,74,133]. On the other hand, the results of our study showed that the quality of the relationships with mothers and fathers exerted a different and peculiar influence on the young adult university students’ level of IA during the COVID-19 pandemic. Indeed, as evidenced above, whereas the relationship between the attachment to fathers and IA was fully mediated by the peritraumatic distress levels, the attachment to mothers exerted a key contribution per se on IA. However, our study also showed a significant mediation role of the alexithymia levels and of their predictive role on peritraumatic distress levels in the relationship between attachment to mothers and IA. In line with our findings, the study by Trumello et al. [71] on a sample of late adolescents also found that the quality of the relationship with mothers, but not with fathers, significantly directly and indirectly (i.e., via psychopathological symptoms) predicted a maladaptive use of the internet. A possible explanation for this finding could be the fact that young adults continue to perceive their mothers as the primary caregiver [134,135] and feel more emotionally involved with them than with their fathers [136]. This could also explain the significant association that we found between the attachment to mothers, but not to fathers, and alexithymia, which is in accordance with the previous studies showing that difficulties in emotional regulation are predicted by insecure attachment to parents and especially to mothers [137,138]. However, in support of the international literature underlying the central role that the paternal relationship continues to assume for young adult psychological well-being [139], the influence of the attachment to fathers on IA was exerted through its predictive role on peritraumatic distress symptoms. On the other hand, as our results also suggested, during young adulthood the influence of peers on an individual’s psychological well-being becomes increasingly important [140]. Indeed, we found that the direct effects of attachment to peers on levels of alexithymia, peritraumatic distress, and IA were all significant, as were the indirect paths via the simple mediation of both alexithymia and peritraumatic distress and the multiple serial mediation of alexithymia and peritraumatic distress. These findings are in line with previous studies evidencing that the insecurity of attachment to peers may predispose young adults to higher difficulties in identifying and describing feelings [141], psychopathological symptoms [142], and internet-related addiction [133]. Moreover, we found greater effect sizes of the effects of attachment to peers on IA compared to those directly and/or indirectly exerted by the attachment to mothers and to fathers. Consistently, our study supported the evidence that, during young adulthood, the quality of attachment to peers exerts the highest influence on psychopathological outcomes compared to those exerted by attachment to parents [88,143]. The significant contribution played by attachment to peers on IA risk has also been widely evidenced [73,133], but to the best of our knowledge, this is the first study focused on its complex interplay with alexithymia and the peritraumatic distress symptoms resulting from COVID-19 among young adult university students.

### 4.3. Limitations and Strengths

There are a number of limitations to this study. Indeed, the absence of a pre-pandemic assessment and the cross-sectional nature of the study design implies the need to treat with caution the effects that we assumed to be a result of COVID-19. Consequently, further studies using longitudinal design should test the prospective associations that we found. Moreover, we used self-report instruments for the assessment of the young adult university students’ attachment to parents and peers, alexithymia, peritraumatic distress symptoms, and IA. Although we chose instruments that are validated and extensively used in this field, evaluating the young adults’ perceptions of their own psychological functioning may produce biased results. Subsequent studies should therefore use more robust assessment tools in studying these processes. Finally, a convenience sampling technique was used for data collection, limiting the generalizability of our findings. Despite the above limitations, this is the first study that explored the dynamic relationships between young adult university students’ attachment to parents and peers, alexithymia, peritraumatic distress, and IA, evidencing a complex reality on the basis of the different attachment figures. Moreover, we found a high proportion of young adult university students who exceeded the clinical cutoff scores for TAS-20, CPDI, and IAT, supporting the importance of implementing studies on this population to further increase the knowledge on the etiopathogenetic factors and to prevent the short- and long-term consequences.

### 4.4. Implications

The results of this study suggest that prevention and intervention programs focused on the promotion of supportive relationships between young adult university students and parents and peers may help young adults in facing COVID-19, preventing its impact on mental health and IA. The restrictions resulting from the pandemic have resulted in the necessity to promote online interventions among university students [144]. In this context, based on the assumption that attachment security supports young adults in coping with stressful life events (such as the COVID-19 pandemic) and based on the necessity to promote online interventions, attachment-based family interventions that use telehealth services have been demonstrated to reduce the psychopathological impact of the COVID-19 pandemic on young adults [145]. At the same time, our findings underlined the central role assumed by the relationship with peers, supporting the importance of online interventions aimed at implementing peer support, which recent studies have shown to be effective in mitigating the negative effects of the pandemic on young adult university students’ mental health [146]. On the other hand, the prevention focused on early detection and intervention and the ability to recognize and discriminate one’s own and others’ emotions also seem to be necessary to reduce the short- and long-term psychopathological consequences of COVID-19 on IA. 

Overall, the pandemic involved a profound redefinition not only of the way of social life but also of academic life, which changed from its traditional face-to-face learning to online learning, significantly increasing the time required to spend online for educational activities. These changes resulted in a higher risk of IA as well as lower satisfaction related to online learning, which in turn increased the risk of IA [147]. In line with this, in addition to psychological counselling services focused on social support and emotional abilities, higher education institutions need to provide effective online instruction and promote coping skills to prevent the risk of IA in online learning environments. As previous studies in the field have also suggested [148], our findings support the importance of reorganizing online education in the direction of limiting the use of technology devices for activities that do not require their specific contribution and of encouraging social activities.

## 5. Conclusions

Our study has added new evidence on the key role played by young adult university students’ attachment to parents and peers, alexithymia, and peritraumatic distress symptoms in predicting IA during the COVID-19 pandemic. A complex relationship between these variables has also been shown, which varied depending on the specific attachment figure. In particular, our findings have evidenced the specific contributions played by the quality of the relationship with mothers, fathers, and peers in predicting levels of IA during the pandemic directly and/or via alexithymia and the peritraumatic distress symptoms resulting from COVID-19. Interestingly, our results suggested that young adult university students with an insecure attachment to peers are at a higher risk for the negative consequences of the pandemic. This could be due to the fact that they are more likely to substitute their attachment to a material object and to their difficulties in coping with negative emotions, as well as to the subsequent higher psychopathological symptoms that the pandemic has, in turn, exacerbated. As a result, they may tend to excessively use the internet as a strategy to cope with the psychopathological sufferance underlying these difficulties.

## Figures and Tables

**Table 1:** Sample Demographic Characteristics.

Age in Years, M (SD)		23.41 (1.72)
Sex, *n* (%)		
	Male	118 (28.8)
	Female	292 (71.2)
Family structure, *n* (%)		
	Intact	304 (74.1)
	Broken	106 (25.9)
Romantic relationship status, *n* (%)		
	Single	255 (62.2)
	Partnered	116 (28.3)
	Cohabitant	38 (9.3)
	Married	1 (0.2)
Living setup, *n* (%)		
	Living alone	10 (2.4)
	Living with friends/housemates	44 (10.7)
	Living with partner	22 (5.4)
	Living with family members	335 (81.5)
Household income (EUR/year), *n* (%)		
	0–15,000	5 (1.2)
	15,001–28,000	25 (6.1)
	28,001–55,000	96 (23.4)
	55,001–75,000	154 (37.6)
	>75,000	130 (31.7)

**Table 2:** Hours per day spent on the internet by young adult university students for leisure activities/entertainment and for study/academic activities before and since the beginning of the COVID-19 pandemic.

Hours Per Day	Before the COVID-19 Pandemic	Since the Beginning of the COVID-19 Pandemic
Leisure activities/entertainment, n (%)		
<2 h	83 (20.2)	34 (8.3)
2–4 h	219 (53.4)	102 (24.9)
4–6 h	90 (22)	157 (38.3)
>6 h	18 (4.4)	117 (28.5)
Study/academic activities, n (%)		
<2 h	213 (52)	49 (12)
2–4 h	119 (29)	80 (19.5)
4–6 h	52 (12.7)	138 (33.7)
>6 h	26 (6.3)	143 (34.9)

**Table 3:** Univariate results of differences between male and female scores on CDPI, TAS-20, and IAT.

	Male M (SD)	Female M (SD)	F_1408_	*p*-Value
CPDI	35.55 (13.82)	40.84 (14.55)	11.425	<0.001
TAS-20	52.83 (12.15)	50.79 (13.73)	1.970	0.16
IAT	30.17 (15.09)	27.97 (15.78)	1.680	0.19

Note: CPDI = COVID-19 Peritraumatic Distress Index; TAS-20 = Total scale of the Toronto Alexithymia Scale; IAT = Internet Addiction Test.

**Table 4:** Pearson correlation coefficients between the starting theoretical model variables.

	1.	2.	3.	4.	5.	6.	7.	8.
1. Sex	1							
2. Age	−0.09 *	1						
3. IPPA mother	0.08	0.01	1					
4. IPPA father	−0.04	−0.04	0.46 **	1				
5. IPPA peer	0.08	0.12 *	0.31 **	0.20 **	1			
6. CPDI	0.16 **	−0.21 **	−0.23 **	−0.30 **	−0.34 **	1		
7. TAS	−0.06	−0.22 **	−0.22 **	−0.10 *	−0.38 **	0.43 **	1	
8. IAT	−0.06	−0.15 **	−0.27 **	−0.21 **	−0.40 **	0.56 **	0.46 **	1

Note: IPPA = Inventory of Parent and Peer Attachment; IPPA mother = Attachment to mother; IPPA father = Attachment to father; IPPA peers = Attachment to peers; CPDI = COVID-19 Peritraumatic Distress Index; TAS-20 = Total scale of the Toronto Alexithymia Scale; IAT = Internet Addiction Test. * *p* < 0.05; ** *p* < 0.01.

**Table 5:** Indirect effects of young adult university students’ attachment to mothers, fathers, and peers on Internet Addiction through alexithymia and peritraumatic distress due to COVID-19.

Indirect Effect	Effect (BootSE)	LLCI	ULCI
IPPA mother → TAS → IAT	0.02 (0.01)	−0.04	−0.001
IPPA mother → CPDI → IAT	−0.01 (0.01)	−0.04	0.01
IPPA mother → TAS → CPDI → IAT	−0.01 (0.01)	−0.02	−0.001
IPPA father → TAS → IAT	0.001 (0.01)	−0.02	0.02
IPPA father → CPDI → IAT	−0.07 (0.01)	−0.10	−0.03
IPPA father → TAS → CPDI → IAT	0.001 (0.01)	−0.01	0.01
IPPA peer → TAS → IAT	−0.05 (0.01)	−0.09	−0.01
IPPA peer → CPDI → IAT	−0.05 (0.01)	−0.08	−0.01
IPPA peer → TAS → CPDI → IAT	−0.03 (0.01)	−0.04	−0.01

Note: all path coefficients are standardized; IPPA = Inventory of Parent and Peer Attachment; IPPA mother = Attachment to mother; IPPA father = Attachment to father; IPPA peers = Attachment to peers; CPDI = COVID-19 Peritraumatic Distress Index; TAS-20 = Total scale of the Toronto Alexithymia Scale; IAT = Internet Addiction Test. BootSE = Boot-strapped standard error; LLCI = Lower level confidence interval; ULCI = Upper level confidence interval.

## Data Availability

The data presented in this study are openly available in FigShare at https://doi.org/10.6084/m9.figshare.21353961.
